# miR-339 Promotes Hypoxia-Induced Neuronal Apoptosis and Impairs Cell Viability by Targeting FGF9/CACNG2 and Mediating MAPK Pathway in Ischemic Stroke

**DOI:** 10.3389/fneur.2020.00436

**Published:** 2020-06-10

**Authors:** Xiao-Zeng Gao, Ru-Hua Ma, Zhao-Xia Zhang

**Affiliations:** ^1^Department of Anesthesiology, North China University of Science and Technology, Tangshan, China; ^2^Emergency Department, Rizhao Hospital of Traditional Chinese Medicine, Rizhao, China; ^3^Department of Geriatrics, Shanxian Central Hospital, Heze, China

**Keywords:** ischemic stroke, miR-339, oxygen-glucose deprivation/reoxygenation (OGD/R), FGF9/CACNG2, apoptosis, MAPK pathway

## Abstract

Ischemic stroke (IS) is a common cerebrovascular disease characterized by insufficient blood blow to the brain and the second leading cause of death as well as disability worldwide. Recent literatures have indicated that abnormal expression of miR-339 is closely related to IS. In this study, we attempted to assess the biological function of miR-339 and its underlying mechanism in IS. By accessing the GEO repository, the expression of miR-339, FGF9, and CACNG2 in middle cerebral artery occlusion (MCAO) and non-MCAO was evaluated. PC12 cells after oxygen-glucose deprivation/reoxygenation (OGD/R) treatment were prepared to mimic *in vitro* the IS model. The levels of miR-339, FGF9, CACNG2, and MAPK-related markers were quantitatively measured by qRT-PCR and Western blot. CCK-8 and flow cytometry analyses were performed to examine cell viability and apoptosis, respectively. IS-related potential pathways were identified using KEGG enrichment analysis and GO annotations. Bioinformatics analysis and dual-luciferase reporter assay were used to predict and verify the possible target of miR-339. Our results showed that miR-339 expression was significantly increased in MCAO and OGD/R-treated PC12 cells. Overexpression of miR-339 inhibited cell viability of PC12 cells subjected to OGD/R treatment. FGF9 and CACMG2 are direct targets of miR-339 and can reverse the aggressive effect of miR-339 on the proliferation and apoptosis of OGD/R-treated PC12 cells. Moreover, miR-339 mediated the activation of the MAPK pathway, which was inhibited by the FGF9/CACNG2 axis in PC12 cells treated by OGD/R stimulation. In summary, these findings suggested that miR-339 might act as a disruptive molecule to accelerate the IS progression via targeting the FGF9/CACNG2 axis and mediating the MAPK pathway.

## Introduction

Ischemic stroke (IS) is a common neurological disease with high recurrence or disability rate (1, 2). The prevalent symptoms of IS are sudden numbness or weakness of face, arm, or leg; temporary confusion; and trouble walking or loss of balance (1, 3). Increasing statistical evidence has demonstrated that IS accounts for 80% of all strokes and seriously damages human health and life (4). Currently, the most efficacious treatment of IS is thrombolysis with reperfusion at the appropriate time (5). However, this treatment tends to induce cerebral ischemia reperfusion injury through enforcing inflammation and glutamate excitotoxicity (6, 7). Therefore, it is of great significance to decipher potential therapeutic targets and effective treatment options for IS.

microRNAs (miRNAs) have been identified as important regulators to participate in various kinds of diseases. Furthermore, the specific effects of several miRNAs in IS have also been elucidated. For example, miR-384-5p can contribute to endothelial progenitor cell viability and angiogenesis in cerebral IS via delta-like ligand 4-mediated Notch signaling pathway (8). Peng et al. indicated that the progression of IS is driven by miR-221 through mediating PTEN/PI3K/AKT signaling pathway (9). The OGD/R-induced neuronal injury can be alleviated by miR-340-5p through activating PI3/AKT pathway (10). These researches greatly support the point that miRNAs are involved in the progression of IS. Recently, a published investigation suggested that miR-339 presents an early and sustained increase in ischemic models of stroke (11). Altintas et al. discovered that aberrant expression of cerebral miRNAs including miR-339 exert the neuroprotective effect against transient cerebral ischemia in diabetic rats (12). Thus, we speculated that miR-339 might play an important role on the development of IS, but the detailed function and the possible mechanism of it in IS remain unclear.

In this present study, the expression of miR-339 in middle cerebral artery occlusion (MCAO) was determined based on the Gene Expression Omnibus (GEO) repository and OGD/R was used to mimic *in vitro* the IS model in PC12 cells. The effect of miR-339 on cell viability and apoptosis of PC12 cells treated by OGD/R and its underlying mechanism was investigated using functional experiments. This exploration may advance both our knowledge of the IS pathogenesis and possible effective treatment agents.

## Materials and Methods

### Clinical Samples Collection

GEO (http://www.ncbi.nlm.nih.gov/geo) is a public functional genomics data repository and helps user query and download experiments and curated gene expression profiles. The two arrays including GSE29287 and GSE61616 were derived from GEO to be used to analyze the expression levels of subjects.

### Cell Culture and OGD/R Model

Rat adrenal medulla-derived pheochromocytoma cell line PC12 was obtained from the American Type Culture Collection (ATCC; Manassas, VA, USA) and maintained in Dulbecco's minimal Eagle's medium (DMEM) containing 10% FBS, 100 mg/ml streptomycin, and 100 U/ml penicillin at 37°C in a conventional atmosphere of 95% O_2_ and 5% CO_2_. The culture medium was changed every 2–3 days. Briefly, to establish the *in vitro* OGD/R model, PC12 cells were incubated in glucose-free DMEM after washing with glucose-free Earle's balanced salt solution and immediately transferred into the anaerobic chamber (1% O_2_, 94% N_2_, and 5% CO_2_) for 2 h. Then, these cells were seeded in the normal medium supplemented with 10% FBS to be incubated for an additional 12 h. In addition, cells of the control group were continuously cultured in the normal condition.

### Transfection

PC12 cells were transfected with specific productions using Lipofectamine 2000 (Invitrogen, Carlsbad, CA, USA) according to the manufacturer's instruction. The productions contain miR-339 mimic, miR-339 inhibitor, and their corresponding negative control (miR-NC), pcDNA3.1-FGF9, pcDNA3.1-CACNG2 and pcDNA3.1 empty vector, si-FGF9 (5′-GACTGGATTTCACTTAGAAATCT-3′), si-CACNG2 (5′-TGGGTGTTTATATAATGAAGAAT-3′), and si-con (5′-AATTCTCCGAACGTGTCACGT-3′). They were all synthesized by GenePharma Co., Ltd (Shanghai, China). At 48-h post transfection, transfectants were exposed to OGD/R treatment and collected to perform further experiments the next day.

### Cell Viability Analysis

CCK-8 assay was conducted to measure the proliferation capacity of PC12 cells stimulated with OGD/R under various transfection. First, cells (1000 cells/well) were inoculated in a 96-well-plate and cultivated for 0, 24, 48, and 72 h. Ten microliters of CCK-8 reagent was subsequently added to every well for an additional 1.5-h incubation at 37°C. Finally, the measurements of optical density (OD) values were finished at 450 nm using the microplate reader to plot the proliferation curve.

### Apoptosis Assay

Following OGD/R treatment and specific transfections, PC12 cells were harvested and suspended using pre-cooled PBS as well as 1 × binding buffer. Then, 100 μl of cell suspension (1–5 × 10^6^/ml) was incubated with 5 μl of Annexin V/FITC for 5 min in the dark. Subsequently, cells were stained with 10 μl of PI and apoptosis rate was analyzed by a flow cytometer (Beckman Coulter, USA) and FlowJo (version 7.6.1; FlowJo LLC) software.

### Luciferase Activity Assay

The fragments of FGF9/CACNG2-wild type (WT) and FGF9/CACNG2-mutant (MUT) carrying miR-339 binding site or not were amplified into pmiR-RB-REPORT™ (RiboBio Co Ltd., Guangzhou, China) for luciferase activity detection. PC12 cells were co-transfected with FGF9/CACNG2-WT or FGF9/CACNG2-MUT and miR-339 mimic/inhibitor as well as miR-NC using Lipofectamine 2000 (Invitrogen). After 48-h transfection, relative luciferase activity was evaluated using Dual-Luciferase Reporter Assay Kit (Promega, Madison, WI, USA) in accordance with the guideline for users.

### qRT-PCR

TRIzol solution was utilized to isolate the total RNA of PC12 cells based on the protocols of the manufacturer. For miR-339, cDNA was reverse transcribed with MiScript Reverse Transcription kit (Qiagen, Hilden, Germany). For FGF9/CACNG2, first-strand cDNA was reverse transcribed by PrimeScript RT kit (Takara biomedical Technology Co., Ltd., Beijing, China). Real-time PCR was implemented on an ABI 7900HT real-time PCR system with MiScript SYBR-Green PCR kit (Qiagen) or SYBR Premix Ex Taq II (TaKaRa, Japan) under indicated conditions (5 min at 95°C followed by 40 cycles of 30 s at 95°C and 45 s at 60°C). All primers were purchased from GenePharma Co., Ltd (Shanghai, China):

miR-339F: 5′-TCCCTGTCCTCCAGGAGCTC-3′,
R: 5′-GAACATGTCTGCGTATCTC-3′;
FGF9F: 5′-CAGCTCCACTGTTGCCAAAC-3′,
R: 5′-ATACAGCTCCCCCTTCTCGT-3′;
CACNG2F: 5′-AATACTCTGCGGTGTCAGCC-3′,
R: 5′-AATACTCTGCGGTGTCAGCC-3′;
U6F: 5′-CTCGCTTCGGCAGCACA-3′,
R: 5′-AACGCTTCACGAATTTGCGT-3′;
GAPDHF: 5′-TGATGGGTGTGAACCACGAG-3′,
R: 5′-AGTGATGGCATGGACTGTGG-3′.

The expression of miR-339 and FGF9/CACNG2 was normalized to U6 or GAPDH using the 2^−ΔΔCT^ method for quantification.

### Western Blotting

PC12 cells were solubilized in RIPA buffer with protease inhibitor, and the total proteins were extracted. Separated proteins were firstly denatured at 95°C for 10 min and quantified by BCA method. Next, denatured proteins (20 μg) were subjected to the 12% SDS-PAGE and transferred to PVDF membranes (Roche, Basel, Switzerland). Five percentage fat-free milk was used to block the PVDF membranes for 1 h at room temperature. Then, PVDF membranes were incubated with indicated primary antibodies (1:1000; Abcam, Cambridge, UK) against FGF9, CACNG2, p-P38 MAPK, P38 MAPK, p-JNK, JNK, and GAPDH at 4°C overnight. After washing with TBST (3 × 5 min), PVDF membranes were probed with horseradish peroxidase (HRP)-labeled secondary antibody for 1 h at room temperature. The protein bands were visualized using ECL solution, and QUANTITY ONE software was employed to measure the gray values of protein bands.

### Statistical Analysis

All statistical analyses were applied using SPSS22.0 (SPSS, Chicago, IL, USA) and GraphPad Prism 6.0 (GraphPad Software, Inc., La Jolla, CA, USA). A Student's *t*-test was conducted to assess the differences of two groups, while the significant differences among multiple groups were performed by one-way ANOVA with Dunnett or Bonferroni *post-hoc* test. KEGG and GO enrichment analyses were conducted according to the criterion of FDR < 0.05. The threshold for statistical significance was considered as *P* < 0.05.

## Results

### miR-339 Promotes OGD/R-Induced Injury in PC12 Cells

**Figure 1:**
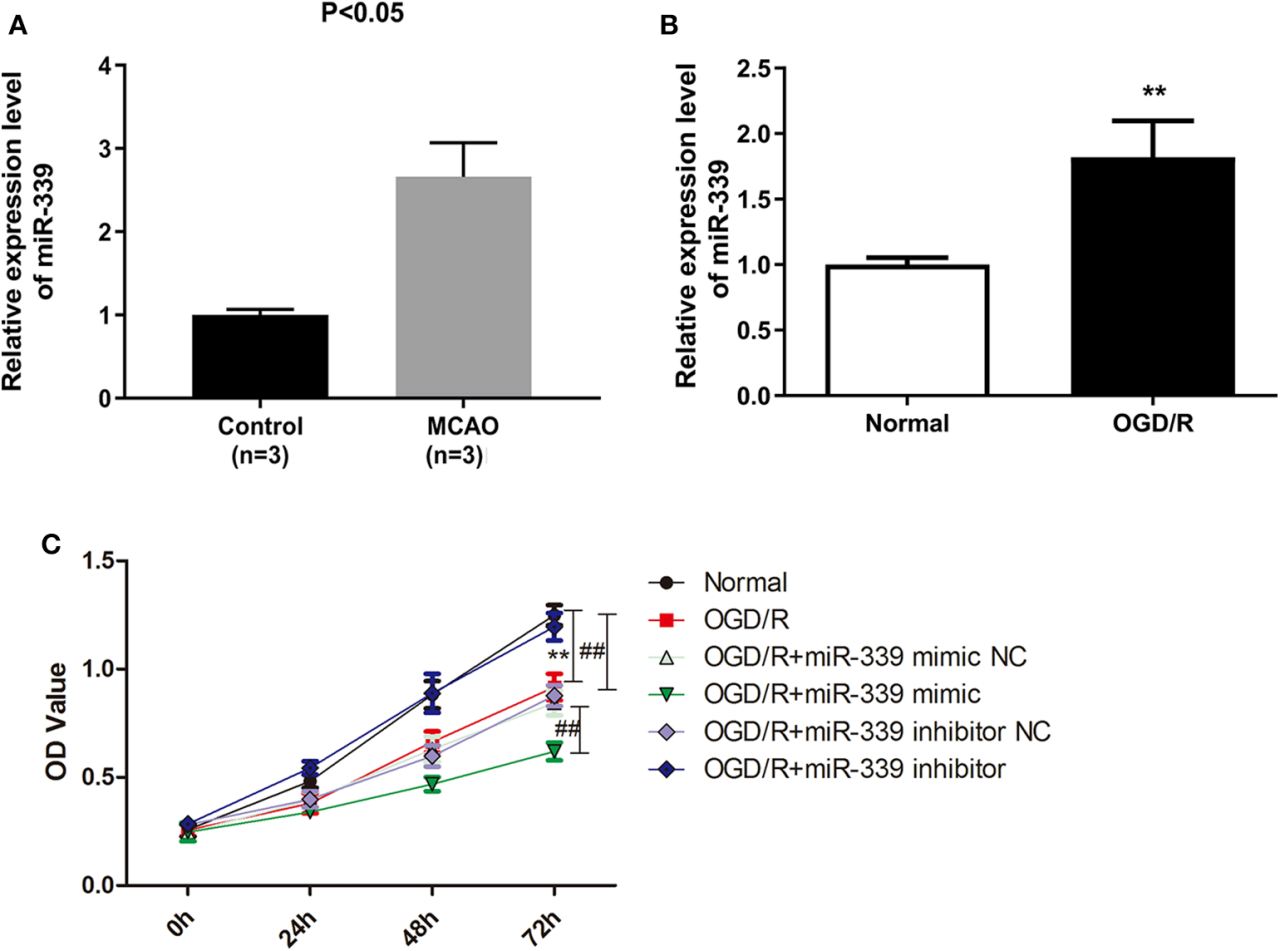
miR-339 plays an aggressive role on IS progression. **(A)** Analysis of miR-339 expression in MCAO patients (*n* = 3) and normal samples (*n* = 3) on the basis of GEO database. Data were presented as the mean (SD), *P* < 0.05 *vs*. control group. **(B)** Measurement of miR-339 expression in PC12 cells after OGD/R treatment using qRT-PCR. Data were presented as the mean (SEM), *n* = 3, ^**^P < 0.01 *vs*. normal group. **(C)** CCK-8 assay was performed to detect the proliferation of OGD/R-induced PC12 cells with different transfections, Data were presented as the mean (SEM), *n* = 3, ^**^P < 0.01 vs. normal group, ^##^*P* < 0.01 vs. OGD/R group.

To inquire the biological function of miR-339 in IS, we firstly analyzed the expression of miR-339 in MCAO and normal tissues based on the GEO datasets. miR-339 was expressed at a higher level in MCAO samples compared with the control (Figure 1A, *P* < 0.05). To mimic *in vitro* the IS model, PC12 cells were stimulated with OGD/R. As shown in Figure 1B, in contrast to the normoxic control, the expression of miR-339 was up-regulated in PC12 cells with OGD/R treatment (*P* < 0.01). Subsequently, we performed CCK-8 test to explore whether miR-339 can regulate the cell viability of OGD/R-induced PC12 cells. The expression of miR-339 in PC12 cells was significantly increased or decreased by the transfection of miR-339 mimic or miR-339 inhibitor, respectively. Results indicated that the OGD/R treatment significantly attenuated the proliferation of PC12 cells; up-regulation of miR-339 further induced a decrease in the proliferation of PC12 cells after OGD/R stimulation compared with miR-339 mimic NC group, whereas down-regulation of miR-339 remarkably elevated the proliferative ability of PC12 cells (Figure 1C, *P* < 0.01). Altogether, these findings demonstrated that overexpression of miR-339 might aggravate the OGD/R-induced injury.

### miR-339 Directly Targets FGF9 and CACNG2 in IS

**Figure 2:**
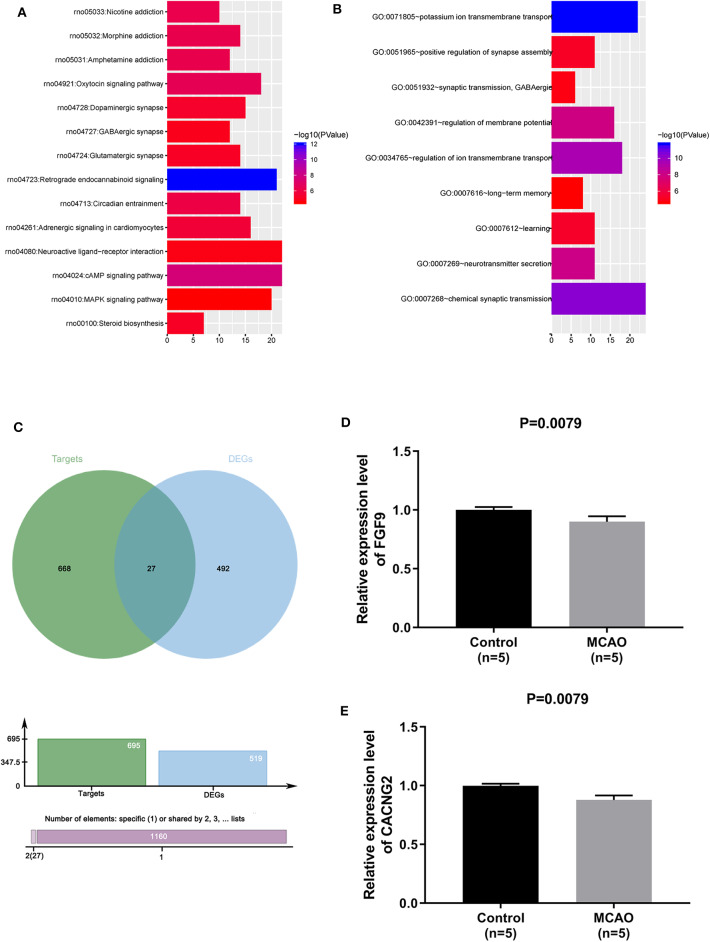
FGF9 and CACNG2 might serve as the potential targets of miR-339 in IS. **(A)** KEGG enrichment analysis and **(B)** GO annotations were conducted to identify the pathways related with down-regulated genes of IS. **(C)** Venn curve of the interaction between the putative targets that predicted by TargetScan and down-regulated genes. **(D)** FGF9 and **(E)** CACNG2 expressions were determined owing to the GEO datasets (accession number: GSE61616, includes five normal controls and five MCAO cases), *P* = 0.0079.

To identify the potential targets of miR-339 in IS, the GSE61616 dataset was downloaded to screen out the DEGs of IS and a total of 1,144 DEGs were identified including 1,046 up-regulated genes and 98 down-regulated genes. Then, the down-regulated genes were analyzed for GO annotation and KEGG pathway enrichment in the David database. Under the condition of FDR <0.05, a total of 14 meaningful pathways and 9 GO-BP were obtained (Figures 2A,B). Prediction tool Targetscan was used to predict the putative targets of miR-339 and 695 potential target genes were finally obtained. By intersecting with the putative targets and down-regulated genes, a total of 27 common genes was achieved (Figure 2C). In addition, given the results of KEGG enrichment analysis, we found that FGF9 and CACNG2 were all enriched in the MAPK signaling pathway; thus, FGF9 and CACNG2 were selected as target genes of miR-339 for further research. Furthermore, data derived from GEO database revealed that the down-regulation of EGF9 and CACNG2 was expressed in MCAO in comparison with that of normal specimens (Figures 2D,E, *P* < 0.01).

**Figure 3:**
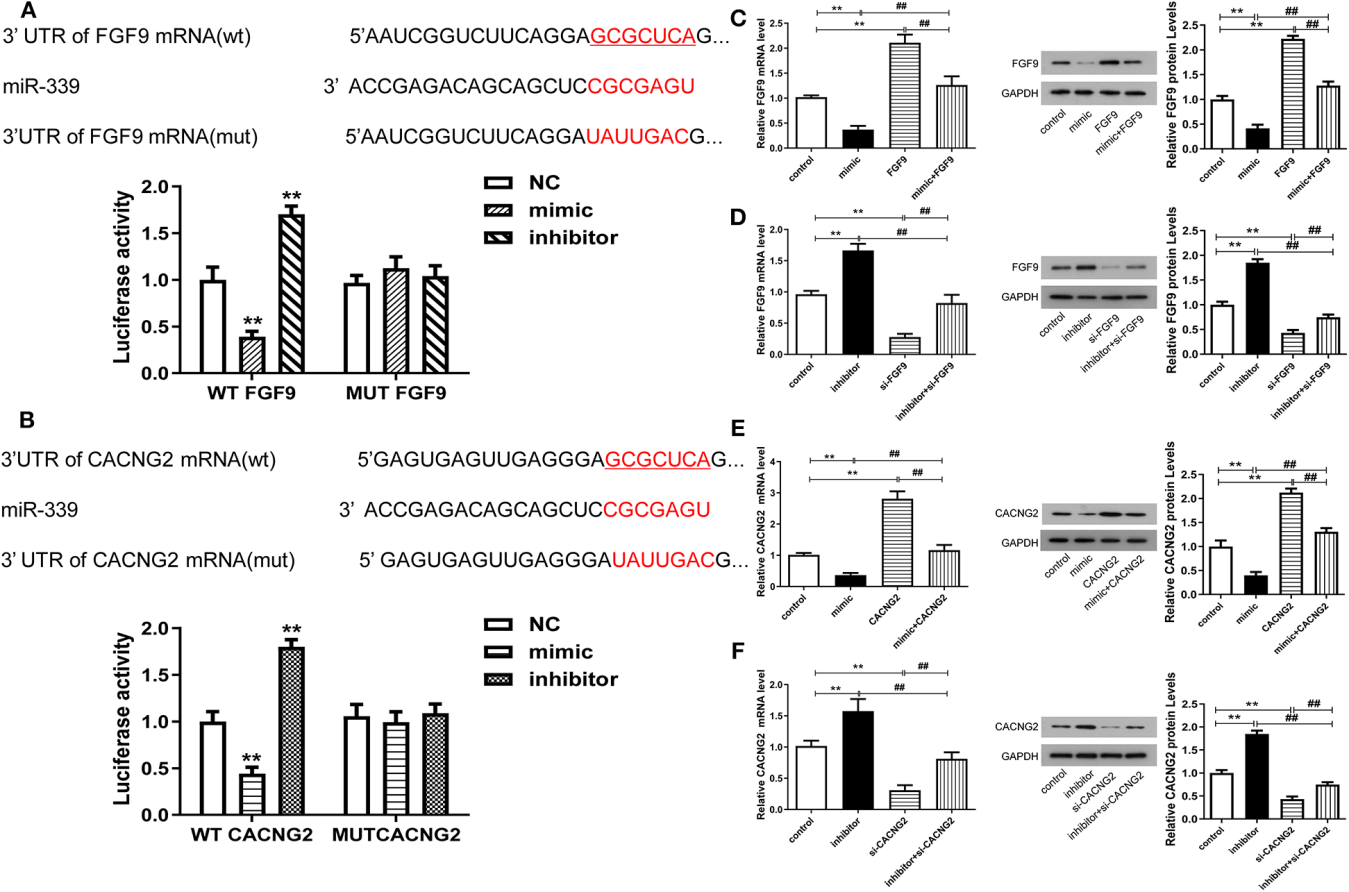
miR-339 can negatively regulate the expression levels of FGF9 and CACNG2. **(A)** The predicted binding site between miR-339 and FGF9. The luciferase activity assay was conducted to examine the luciferase activity of WT FGF9 and MUT FGF9 in PC12 cells transfected with miR-339 mimic or miR-339 inhibitor. Data were presented as the mean (SEM), *n* = 3, ^**^P < 0.01 vs. negative control (NC) group. **(B)** Sequence of interaction site between miR-339 and CACNG2. Dual-luciferase reporter assay was implemented to assess the relative luciferase activity of WT CACNG2 and MUT CACNG2 in PC12 cells after miR-339 mimic or miR-339 inhibitor transfection. Data were presented as the mean (SEM), *n* = 3, ^**^P < 0.01 vs. NC group. **(C,D)** Detection of FGF9 mRNA and protein levels in PC12 cells with various transfections, including blank control, miR-339 mimic/inhibitor, pcDNA3.1-FGF9/si-FGF9, mimic+FGF9/inhibitor+si-FGF9. Data were presented as the mean (SEM), *n* = 3, ^**^P < 0.01 vs. control group, ^##^*P* < 0.01 vs. mimic+FGF9/inhibitor+si-FGF9. **(E,F)** Exploration of CACNG2 mRNA and protein levels in PC12 cells with different transfections, including blank control, miR-339 mimic/inhibitor, pcDNA3.1-CACNG2/si- CACNG2, mimic+CACNG2/inhibitor+si-CACNG2. Data were presented as the mean (SEM), *n* = 3, ^**^P < 0.01 vs. control group, ^##^*P* < 0.01 vs. mimic+CACNG2/inhibitor+si-CACNG2.

To further validate the correlation between miR-339 and FGF9/CACNG2, dual-luciferase reporter gene assay was conducted and results displayed that the luciferase activity of the WT FGF9 group was significantly changed (decreased after miR-339 mimic transfection and increased after miR-339 inhibitor transfection) (Figure 3A, *P* < 0.01). However, there was no significant difference in the MUT FGF9 group. The consistent results were observed in WT CACNG2 and MUT CACNG2 groups (Figure 3B, *P* < 0.01). In addition, qRT-PCR and Western blot were performed to assess whether abnormal expression of miR-339 affects the expression of FGF9 or CACNG2 in PC12 cells. As the results presented, FGF9 and CACNG2 expression were negatively regulated by the expression of miR-339 (Figures 3C–F, *P* < 0.01). Compared with the control group, PC12 cells transfected with miR-339 mimic showed a decrease level of FGF9 expression while the co-transfection of miR-339 mimic and pcDNA3.1-FGF9 partially recovered the mimic-induced down-regulation of FGF9. miR-339 inhibitor significantly increased the expression of FGF9 and the addition of si-FGF9 inhibited the promoting effect of miR-339 inhibitor on FGF9 expression. Similarly, CACNG2 expression was also repressed by miR-339 mimic and elevated attributed to the transfection of miR-339 inhibitor. Furthermore, the effect of miR-339 mimic and inhibitor on CACNG2 expression could be reversed by CACNG2 or si-CACNG2, respectively. Collectively, all data suggested that FGF9/CACNG2 were direct targets of miR-339 in IS.

### miR-339 Inhibits Cell Proliferation and Induces Apoptosis of OGD/R-Treated PC12 Cells Through Directly Targeting FGF9 and CACNG2

**Figure 4:**
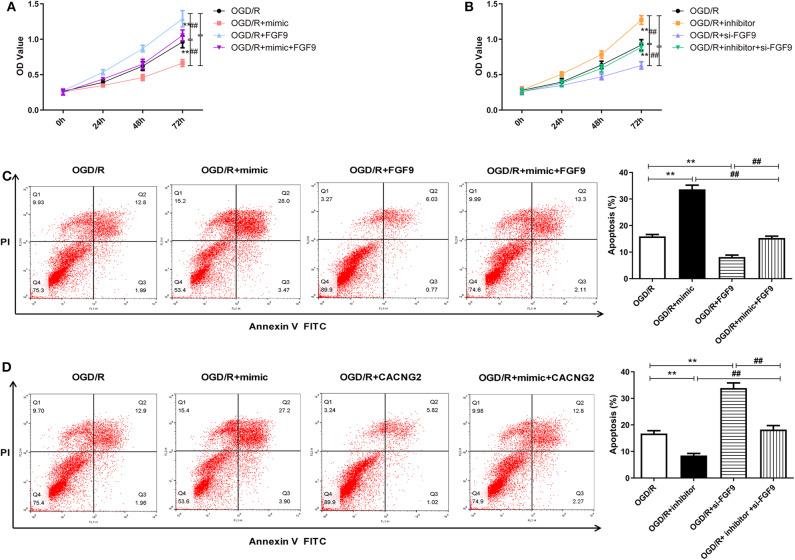
miR-339 aggravated OGD/R-induced injury through targeting FGF9 in PC12 cells. **(A,B)** CCK-8 assays were performed to detect the PC12 cell viability after OGD/R treatment and different transfections. Data were presented as the mean (SEM), *n* = 3, ^**^P < 0.01 vs. OGD/R group, ^##^*P* < 0.01 vs. OGD/R+mimic+FGF9 or OGD/R+inhibitor+si-FGF9 group. **(C,D)** Annexin V-fluorescein isothiocynate (FITC)/propidium iodide (PI) staining and flow cytometry analysis were implemented to evaluate cell apoptosis. Data were presented as the mean (SEM), *n* = 3, ^**^P < 0.01 vs. OGD/R group, ^##^*P* < 0.01 vs. OGD/R+mimic+FGF9 or OGD/R+inhibitor+si-FGF9 group.

**Figure 5:**
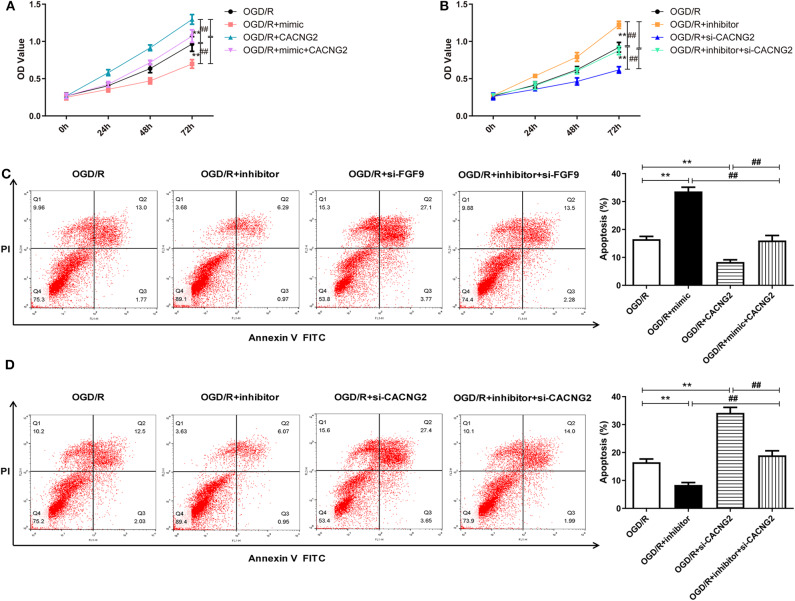
The regulation of miR-339 on OGD/R-induced PC12 cell injury was modulated by CACNG2. **(A,B)** After transfection with various agents, the proliferative ability of PC12 cells stimulated by OGD/R was determined. Data were presented as the mean (SEM), *n* = 3, ^**^P < 0.01 vs. OGD/R group, ^##^*P* < 0.01 vs. OGD/R+mimic+CACNG2 or OGD/R+inhibitor+si-CACNG2 group. **(C,D)** The apoptotic potential of OGD/R-treated PC12 cells was measured using flow cytometry analysis. Data were presented as the mean (SEM), *n* = 3, ^**^P < 0.01 vs. OGD/R group, ^##^*P* < 0.01 vs. OGD/R+mimic+CACNG2 or OGD/R+inhibitor+si-CACNG2 group.

In order to explore whether the regulatory effect of miR-339 on IS progression is associated with FGF9/CACNG2, further experiments containing CCK-8 and flow cytometry analyses were conducted. Interestingly, overexpression of miR-339 intensified the OGD/R-induced injury; up-regulation of FGF9/CACNG2 protected PC12 cells from OGD/R-induced damage; co-transfection of miR-339 mimic and FGF9/CACNG2 recovered cell viability of PC12 cells to normal levels (Figures 4A, 5A, *P* < 0.01). Apoptosis analysis revealed that miR-339 enhancement could promote the apoptosis rate of PC12 cells after OGD/R treatment, while overexpression of FGF9 or CACNG2 significantly inhibited apoptosis. By contrast, co-transfection of miR-339 mimic and FGF9 or CACNG2 recovered the apoptotic ability to the conventional level (Figures 4C, 5C, *P* < 0.01). On the contrary, the transfection of miR-339 inhibitor protected PC12 cells from OGD/R treatment, contributed to cell proliferation, and suppressed apoptosis. The knockdown of FGF9 or CACNG2 exhibited a converse role on PC12 cells after OGD/R injury. The co-transfection of miR-339 inhibitor and si-FGF9 or si-CACNG2 overturned their individually effect on cell proliferation as well as apoptosis (Figures 4B,D, 5B,D, *P* < 0.01). These results determined that miR-339 aggravated OGD/R-stimulated cell damage via inhibiting the expression of FGF9/CACNG2 in PC12 cells.

### The miR-339/FGF9/CACNG2 Axis Regulates the MAPK Signaling Pathway in OGD/R-Induced PC12 Cells

**Figure 6:**
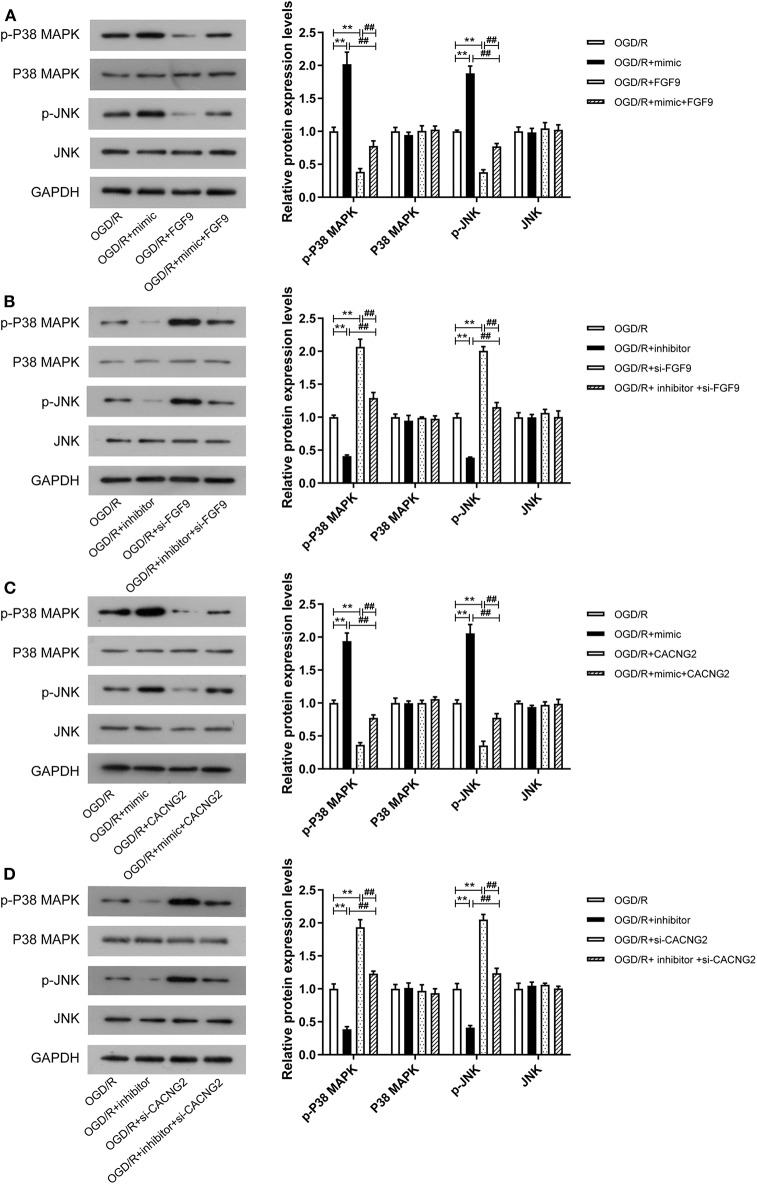
Effects of miR-339 on the MAPK pathway were associated with FGF9/CACNG2 in OGD/R-stimulated PC12 cells. **(A–D)** The immunoblots of p-P38 MAPK, P-38 MAPK, p-KNK, and JNK (left panels), and quantification of the gray values of corresponding protein bands (right panels) in PC12 cells after OGD/R treatment. Data were presented as the mean (SEM), *n* = 3, ^**^P < 0.01 vs. OGD/R group, ^##^*P* < 0.01 vs. OGD/R+mimic+FGF9/CACNG2 or OGD/R+inhibitor+si-FGF9/si-CACNG2 group.

Considering that the FGF9 and CACNG2 were all enriched in the MAPK pathway (based on the KEGG enrichment analysis), we speculated that the effect of miR-339/FGF9/CACNG2 in IS may be correlated with the activity of the MAPK pathway. To clarify this potential molecular mechanism, the protein levels of key markers implicated in the MAPK pathway were measured by Western blotting including p-P38, P38, p-JNK, and JNK. As shown in Figures 6A,C, in the OGD/R+mimic group, the protein levels of p-P38 and p-JNK were significantly increased compared with the OGD/R group (*P* < 0.01). Overexpression of FGF9 or CACNG2 led to a remarkable decrease in the expression level of p-P38 and p-JNK when compared with the OGD/R group (*P* < 0.01). Furthermore, the co-transfection of miR-339 mimic and FGF9 or CACNG2 recovered the effects of either miR-339 mimic or FGF9/CACNG2 on the expression levels of p-P38 and p-JNK. Compared with the OGD/R group, miR-339 inhibitor induced the reduction of p-P38 and p-JNK expression while silencing of FGF9 or CACNG2 elevated the protein levels of p-P38 and p-JNK. A significant restoration of the attenuation or increase in the protein levels of p-P38 and p-JNK was induced by miR-339 inhibitor or si-FGF9/CACNG2, respectively (Figures 6B,D, *P* < 0.01). The results also exhibited that there were no significant changes in the P38 and JNK expression after different treatments (Figure 6, *P* < 0.01). In summary, these data indicated that miR-339 could activate the MAPK pathway through inhibiting the FGF9 and CACNG2 expression in IS development.

## Discussion

As previously described, cerebral ischemia/reperfusion (I/R) injury is the leading cause of cerebrovascular diseases and the frequent kind being stroke (13). It is well-known that the mechanism of cerebral I/R injury is complex, which refers to multiple biological processes, including apoptosis, inflammation, and necrosis (14, 15). Therefore, it is an urgent task to explain the potential mechanism of cerebral I/R injury that mainly induces IS. OGD/R treatment has been widely used to mimic the cell state after I/R injury *in vitro* (16). Our present study demonstrated that miR-339 expression was markedly increased in MCAO. By constructing the OGD/R model, we also found that the expression of miR-339 was elevated in PC12 cells. Through *in vitro* functional analyses, miR-339 could accelerate the progression of IS via targeting FGF9/CACNG2 and mediating the activity of MAPK signaling pathway, indicating that miR-339/FGF9/CACNG2 might act as novel therapeutic targets to improve the grave outcomes of patients with IS.

Accumulating studies have shown that dysregulation of miR-339 is associated with a variety of cancers. Yu et al. have reported that miR-339 inhibits the invasion and migration of pancreatic tumor cells through down-regulating ZNF689 expression (17). Similarly, knockdown of miR-339 in hepatocellular carcinoma cells promotes proliferation and invasion and suppresses apoptosis (18). On the contrary, miR-339 exerts an opposite role to cancer in other diseases. Hu et al. demonstrated that miR-339 is up-regulated in stem cell leukemia/lymphoma syndrome and promotes the development of stem cell leukemia/lymphoma syndrome via suppressing BCL211 and BAX expression (19). Importantly, Dhiraj et al. have profiled miRNAs in the whole ischemic infarct and identified that miR-339 may serve as an essential factor and modulates the salvageable ischemic penumbra-related cellular pathways (11). Consistent with this prior study, miR-339 was found to be overexpressed in MCAO and PC12 cells exposed to OGD/R in our study. Up-regulation of miR-339 could significantly attenuate PC12 cell viability after OGD/R treatment. These data elucidated that miR-339 might aggravate the PC12 cell injury induced by OGD/R.

To explore the mechanism and targets of miR-339 in IS in depth, we screened out the DEGs in MCAO based on GEO repository and predicted the possible target genes of miR-339 using TargetScan website. After enriching DEGs using KEGG and GO analysis, a total of 14 pathways including the MAPK pathway were obtained. A previous study indicated that the MAPK pathway is an important pathway stimulated in the early stage of IS (20). Furthermore, the common genes FGF9 and CACNG2 that were identified by intersecting the DEGs and putative targets of miR-339 were enriched in the MAPK pathway. Thus, FGF9 and CACNG2 were selected as the targets of miR-339 for further detection. FGF9 (fibroblast growth factor 9) is located on the chromosome 13q11-q12 and initially considered as a secreted agent revealing a mitogenic role on the glial cells (21, 22). The secretion of FGF9 can improve the survival rate and neurite growth of SH-SY5Y neuroblastoma cells (23). The miR-182/FGF9 axis is associated with nerve injury-caused phenotype (24). CACNG2 (calcium voltage-gated channel auxiliary subunit gamma 2) has been proven to be a pain susceptibility gene. CACNG2 could encode the gamma-2 transmembrane AMPA receptor protein (TARP) stargazing and is involved in the modulation of neuronal Ca^2+^ channels. It plays a central role in the cerebellar function and epilepsy (25–27). Therefore, we reasonably speculated that FGF9/CACNG2 might participate in the development of IS. In this present study, we found that FGF9/CACNG2 were directly targeted by miR-339 and the overexpression of miR-339 could significantly attenuate the expression of FGF9 and CACNG2 in PC12 cells. Rescue experiments in OGD/R-induced PC12 cells further suggested that the promoting effect of miR-339 on the progression of IS was reversed by the overexpressing FGF9/CACNG2.

The role of the MAPK pathway in the pathogenesis of IS uncovered that regulating the activity of the MAPK pathway through ameliorating or intensifying key markers may be an important event for IS development (28). P38 and JNK have been regarded as key markers in the MAPK signaling pathway, which exert pathological and physiological roles when they are phosphorylated (29, 30). Our results demonstrated that abnormal expression of miR-339 affected the protein levels of p-P38 and p-JNK in OGD/R-induced PC12 cells as well as FGF9/CACNG2. Furthermore, the interferences of FGF9 and CACNG2 could rescue the aggressive impacts of miR-339 enhancement on OGD/R-stimulated injury in PC12 cells. Consequently, we summarized that miR-339 could contribute to the development of IS through targeting FGF9/CACNG2 and mediating the activity of the MAPK pathway.

In conclusion, we observed that overexpression of miR-339 in PC12 cells after OGD/R treatment inhibited cell viability and induced apoptosis via mediating the FGF9/CACNG2 axis and the MAPK pathway. Due to the insufficient number of MCAO, it is difficult to strongly conclude the biological effects of miR-339/FGF9/CACNG2 in IS development. Besides, their specific role still needs to be performed *in vivo*. To sum up, this current study sheds new insights into the mechanism of OGD/R injury and provides promising therapeutic targets for IS treatment.

## Data Availability Statement

Publicly available datasets were analyzed in this study. This data can be found here: GSE29287 and GSE61616.

## Author Contributions

X-ZG and R-HM carried out the research, performed the analysis, and participated in the writing of the manuscript. X-ZG and Z-XZ designed and supervised the research, and participated in the writing and reviewing of the manuscript. All authors have read and approved the final manuscript.

## Conflict of Interest

The authors declare that the research was conducted in the absence of any commercial or financial relationships that could be construed as a potential conflict of interest.
